# Daytime napping, comorbidity profiles, and the risk of sarcopenia in older individuals

**DOI:** 10.3389/fphys.2022.1000593

**Published:** 2022-11-01

**Authors:** Zhigang Hu, Ailan Yang, Yufeng Tian, Xinyu Song

**Affiliations:** ^1^ Department of Respiratory and Critical Care Medicine, The First College of Clinical Medicine Science, China Three Gorges University, Yichang, China; ^2^ Department of Respiratory and Critical Care Medicine, Yichang Central People’s Hospital, Yichang, China; ^3^ Department of Respiratory and Critical Care Medicine, Yichang Central People’s Hospital at Zhijiang, Zhijiang, China; ^4^ Department of Teaching Office, Three Gorges University, Yichang, China

**Keywords:** older adults, daytime napping, sarcopenia, comorbidity, Chinese health and retirement longitudinal study

## Abstract

Appropriate daytime napping is associated with the decreased risk of cerebro-cardiovascular diseases, but whether daytime napping affects sarcopenia remains to be explored. Our study plans to examine the associations between sarcopenia with daytime napping and comorbidity. The study population came from the China Health and Retirement Longitudinal Study 2011–2015. Latent class analysis (LCA) was used to identify comorbidity profiles based on 14 doctor-diagnosed chronic diseases. Subsequently, smooth function and restricted cubic spline with three binomial regression models determined the associations between sarcopenia with daytime napping and comorbidity profiles. About 18.7% (2,894) and 5.4% (832) of 15,404 individuals were diagnosed with sarcopenia and severe sarcopenia. LCA delineated four classes as the best fit as follows: dominant heart diseases or risks (class 1, *N* = 2,203), dominant chronic lung diseases (class 2, *N* = 740), minimal or least diseases (class 3, N = 10,612, reference), and dominant digestive diseases and rheumatism (class 4, N = 1849). Compared with the reference group (class 3), the multivariate-adjusted ORs (95% CIs) of sarcopenia in model 3 were 0.72 (0.60–0.88) for class 1, 1.17 (0.92–1.51) for class 2, and 0.92 (0.77–1.09) for class 4. Smooth function and restricted cubic spline suggested that individuals who napped about 60 min seemingly had the lowest risk of sarcopenia. Individuals who napped for 1–59 min (adjusted OR = 0.80, 95% CI: 0.68–0.94) and 60–119 min (adjusted OR = 0.83, 95% CI: 0.72–0.95) had the significantly lower risk of sarcopenia but not severe sarcopenia than those who did not nap. Insufficient and excessive daytime napping might be associated with the increased risk of sarcopenia, especially in individuals with a dominant chronic lung disease profile.

## Introduction

China has the largest elderly population in the world, and the accelerating process of population aging may be attributed to the increase in longevity and reduction in fertility (Zhao et al., 2014). The proportion of individuals who are aged 60 years will increase from 10% in 2000 to approximately 30% in 2050 (United Nations, 2009). Sarcopenia, with the accelerated loss of muscle mass and function, is an age-related process in older individuals and leads to adverse health outcomes including functional decline, falls, frailty, disability, and mortality (Cruz-Jentoft and Sayer, 2019). The frequently underlying causes of sarcopenia comprise contemporaneous risk factors (such as comorbidity, etc.), lifestyle, and genetic factors operating during the course of life (Cruz-Jentoft and Sayer, 2019). Early identification and intervention of comorbidity and a healthy lifestyle can potentially decrease the risk of developing sarcopenia.

A 12-year longitudinal analysis involving 2,867 individuals suggested that multimorbidity at baseline is associated with an increased risk of developing sarcopenia (Veronese et al., 2021). Compared with no multimorbidity, the increase in medical condition at baseline was positively associated with the increased risk of developing sarcopenia during follow-up (Veronese et al., 2021). Another study reported that the level of comorbidity was not associated with the prevalence of sarcopenia (Volpato et al., 2014). Cruz-Jentoft et al. summarized the risk factors of sarcopenia with relation to comorbidity, which included metabolic cardiorespiratory and neurological disorders, cancer, bone and joint diseases, etc. (Cruz-Jentoft and Sayer, 2019). These studies indicated that not all comorbidities can affect the prevalence of sarcopenia, and different diseases might be associated with different prevalence of sarcopenia. A targeted special comorbidity profile can provide more effective strategies in the early prevention and treatment of sarcopenia.

Daytime napping is believed to be a healthy lifestyle in China. More than 50% of middle-aged and older individuals reported routine napping in the Chinese national representative study (Zhang et al., 2020). Previous studies have demonstrated the beneficial effects of healthy daytime napping on health conditions, immune function, cognitive performance, and memory consolidation (Faraut et al., 2017; Zhang et al., 2020). The possible mechanisms of physiological and cognitive effects of healthy daytime napping included the following pathways (Faraut et al., 2017): (1) change in expression patterns of immune markers (neutrophil counts and IL-6) and recovery of low-grade inflammation secondary to sleep loss, (2) reduced sympathetic nervous activity and decreased release of sympathetic system mediators, thereby reducing the heart rate and blood pressure, and (3) stress-releasing effects (cortisol and catecholamines) to alleviate the vascular neutrophil mobilization. However, long-term daytime napping (e.g., >60 min) was found to be positively associated with adverse cardiovascular and diabetes outcomes, increased mortality, and the reduction of cognitive function in older individuals (Faraut et al., 2017; Zhang et al., 2020). As yet, scant studies estimate the association between daytime napping with the risk of sarcopenia.

Here, we plan to investigate the effects of daytime napping and comorbidity profiles on the prevalence of sarcopenia in older individuals using the China Health and Retirement Longitudinal Study (CHARLS). We want to test the following hypotheses: first, the prevalence of sarcopenia could vary depending on morbidity profiles and second, daytime napping with an appropriate duration might decrease the risk of sarcopenia.

## Materials and methods

### Study design and population

The CHARLS conducted in 2011 is a nationally representative study for individuals aged ≥45 years to analyze aging-related issues and promote interdisciplinary research on aging (Zhao et al., 2014). The CHARLS covered 28 provinces, 150 county-level units, and 450 village-level units. All individuals received a face-to-face computer-assisted personal interview and physical measurements at every 2-year follow-up. New individuals were added to each wave of the CHARLS. As yet, three waves (wave 1 in 2011, wave 2 in 2013, and wave 3 in 2015) of the CHARLS provided data about the physical performance and anthropometric measures. The Biomedical Ethics Review Committee of Peking University approved the CHARLS. The National School of Development of Peking University collected written informed consent of all individuals. More detailed description of the CHARLS has been reported elsewhere (Zhao et al., 2014) and in the following link: http://charls.pku.edu.cn/en/.

### Assessment of sarcopenia

According to previous studies (Wu et al., 2021; Gao et al., 2022), the diagnosis of sarcopenia must fulfill low-appendicular skeletal muscle mass (ASM) and low muscle strength/physical performance. Individuals with low muscle mass, muscle strength, and physical performance were diagnosed with severe sarcopenia. The age cut-offs of sarcopenia were set at 60 years of age in this study. Low muscle strength was measured by handgrip strength (<28.0 kg for men and <18.0 kg for women); meanwhile, gait speed <1.0 m/s and 5-time chair stand test ≥12 s were defined as low physical performance. The following anthropometric equation for the height-adjusted muscle mass (ASM/Ht^2^) was used to determine whether low ASM exists in the Chinese population (Wen et al., 2011; Hu et al., 2017; Yang et al., 2017; Wu et al., 2021; Gao et al., 2022; Hu et al., 2022): ASM/Ht^2^ =(0.193*body weight +0.107* height - 4.157* gender - 0.037* age - 2.631)/height^2^. Similar to previous studies, the cut-off for defining low muscle mass was based on ASM/Ht^2^ of the lowest 20th percentile of the study population. Therefore, ASM/Ht^2^ < 6.87 for men and <5.07 for female were regarded as low ASM in this study.

### Napping duration and comorbidity

The data of daytime napping were based on the following question: During the past month, how long did you take a nap after lunch in general? Daytime napping was divided into four groups: 0 (no napping), 1 to 59, 60 to 119, and ≥120 min.

All individuals were requested to answer whether they experienced 14 doctor-diagnosed chronic diseases (hypertension, dyslipidemia, hyperglycemia, cancer, chronic lung diseases, liver diseases, heart diseases, stroke, kidney diseases, digestive diseases, emotional or psychiatric problems, memory-related diseases, arthritis or rheumatism, and asthma).

### Confounding risk factors

We collected some variables as potentially confounding factors, which are as follows: sex (male vs. female), age (60–69 years vs. 70–79 years vs. ≥ 80 years), region (North vs. East and Central vs. West), urban/rural (urban vs. rural), married status (current unmarried vs. current married), body mass index (BMI), smoking (never vs. ever vs. current), alcohol (never vs. ever vs. current), night sleep duration, peak expiratory flow (PEF), accident (yes vs. no), fallen down (yes vs. no), hip fracture (yes vs. no), and depression.

According to the recommendation of WHO, BMI was divided into the four following groups: underweight (<18.5 kg/m^2^), normal (18.5 to <24.0 kg/m^2^), overweight (24.0 to <28.0 kg/m^2^), and obesity (≥28.0 kg/m^2^) (Li et al., 2020). Referring to a previous study (Wang et al., 2020), night-sleep duration was classified into five groups: <6 h as very short sleep duration, 6–6.99 h as short sleep duration, 7–7.99 h as healthy sleep duration, 8–8.99 h as relatively long sleep duration, and ≥9 h as long sleep duration. Baseline PEF was measured using a peak flow meter (EverpureTM, Shanghai, China) with a disposable mouthpiece in L/min (Ma et al., 2019). PEF could reflect the strength of abdominal muscles. Low PEF indicated the weakness of abdominal muscles, which would limit the ability of physical activity, especially high-intensity physical activity (Ma et al., 2019). In addition, the CHARLS assessed depressive symptoms by the 10-item Center for Epidemiological Studies–Depression Scale (CES-D10). Each item of the CES-D10 comprised four answers, which were charged with scores from 0 to 3. Total scores of CES-D10 ≥ 12 were considered depression with reference to previous studies (Cheng and Chan, 2005; Cheng et al., 2016; Chen et al., 2020).

### Statistical analysis

Statistical analyses of this study included three components. First, the study population was categorized into no sarcopenia, nonsevere sarcopenia, and severe sarcopenia. Chi-square test was used to compare the differences among three groups with categorical variables presented as counts and percentages (%). The authors displayed continuous variables through means and standard deviations and subsequently performed the comparison of the three groups using the Mann–Whitney *U* test for skewed continuous variables and Student’s t test or one-way ANOVA with Dunnett test for normally distributed continuous variables. Second, latent class analysis (LCA) was used to determine the best-fitting latent model and class of comorbidity profiles based on 14 doctor-diagnosed chronic diseases (Bui et al., 2021). Lower values of Aikaike’s information criterion (AIC) and adjusted Bayesian information criterion (aBIC) with higher value of entropy indicated the better fit on condition that the Lo–Mendell–Rubin test (LMRt) and bootstrap likelihood ratio test were less than 0.05. Third, we estimated the associations between daytime napping and comorbidity profiles with the prevalence of sarcopenia using smooth function and restricted cubic spline with three binomial regression models. Model 1 included demographic characteristics (sex, age, region, urban/rural, married status, and body mass index), model 2 added behavioral factors (alcohol, smoking status, night-sleep duration and PEF), and model 3 added behavioral factors and comorbidities (accidents, fallen down, hip fracture, and depression). All statistical analyses were done in Empower(R) (www.empowerstats.com; X&Y solutions, Inc. Boston MA) and Mplus. Odd ratios (ORs) with 95% confidence intervals (CIs) represented the strength of association, and a two-tailed *p* < 0.05 was considered statistically significant.

### Ethical procedures

Because all related data were derived from the open CHARLS, no patient was involved in the recruitment and conduct of the study. This study was deemed exempt for review by the Institutional Review Board at China’s Three Gorges University.

## Results

In three cycles of the CHARLS (2011, 2013, and 2015), a total of 15,404 individuals aged 67.6 ± 6.2 years was incorporated into the study. The majority of older individuals were between 60 and 69 years of age (67.2%), followed by those between 70 and 79 (27.7%) and those of 80 or over (5.1%). The majority were male (51.9%), currently married (81.3%), and residents in rural areas (62.6%). The proportion of individuals who never smoked and drank alcohol was 54.8% and 66.8%, respectively. The mean value of PEF was 276.1 L/min (±120.3 L/min). About 58.9% of older individuals reported daytime napping. The prevalence of sarcopenia and severe sarcopenia was 18.7% and 5.4%, respectively. Individuals with sarcopenia had higher ages and scores of CED-S10 and lower PEF, BMI, and night-sleep duration than those with no sarcopenia. The severity of sarcopenia was negatively associated with PEF and positively associated with age (see Table 1). Post hoc test showed that the mean values of PEF declined by 54.8 L/min (95% CI: 61.1 L/min, -48.6 L/min) in the nonsevere sarcopenia group and 95.6L/min (95% CI: −105.1 L/min, −86.3 L/min) in the severe sarcopenia group compared with individuals without sarcopenia. In comparison to individuals without sarcopenia, CED-S10 scores increased by 0.8 scores (95% CI: 0.5, 1.1) and 0.9 scores (95% CI: 0.5, 1.3) in the nonsevere and severe sarcopenia groups, respectively. The mean values of BMI and night sleep duration also demonstrated the significant reduction in the nonsevere and severe sarcopenia groups than those in individuals without sarcopenia (see Table 1). Aging (see Supplementary Figure S1) and increasing scores of depressive symptoms after 5 scores (see Supplementary Figure S2) were associated with the increasing prevalence of sarcopenia and severe sarcopenia. Detailed characteristics of the study population were shown in Table 1.

**TABLE 1:** Characteristics of the study population in the China Health and Retirement Longitudinal Study.

	No sarcopenia	Nonsevere sarcopenia	Severe sarcopenia	p
N	12,510 (82.3%)	2062 (13.3%)	832 (5.4%)	
Sex				0.084
Male	6,511 (52.0%)	1,033 (50.1%)	453 (54.4%)	
Female	5,999 (48.0%)	1,029 (49.9%)	379 (45.6%)	
Age				<0.01
60–69 years	9,052 (72.4%)	1,053 (51.1%)	248 (29.8%)	
70–79 years	3,044 (24.3%)	827 (40.1%)	398 (47.8%)	
≥80 years	414 (3.3%)	182 (8.8%)	186 (22.4%)	
Mean age	66.7 ± 5.7	70.0 ± 6.7	74.0 ± 7.1	<0.01
Region				<0.01
Southwest	3,399 (27.2%)	816 (39.6%)	302 (36.3%)	
South and central	6,526 (52.2%)	1,014 (49.2%)	414 (49.8%)	
North	2,585 (20.7%)	232 (11.3%)	116 (13.9%)	
Urban/Rural				<0.01
Rural	7,491 (59.9%)	1,534 (74.4%)	624 (75.0%)	
Urban	5,019 (40.1%)	528 (25.6%)	208 (25.0%)	
Married status				<0.01
Current unmarried	2,100 (16.8%)	489 (23.7%)	285 (34.3%)	
Current married	10,410 (83.2%)	1,573 (76.3%)	547 (65.7%)	
Body mass index category (BMI)				<0.01
Underweight	77 (0.6%)	833 (40.4%)	344 (41.3%)	
Normal	6,343 (50.7%)	1,228 (59.6%)	486 (58.4%)	
Overweight	4,463 (35.7%)	1 (0.0%)	2 (0.2%)	
Obesity	1,627 (13.0%)	0 (0.0%)	0 (0.0%)	
Mean BMI(kg/m^2^)	24.4 ± 4.0	18.7 ± 1.5	18.8 ± 1.8	<0.01
Smoking				<0.01
Never	6,979 (55.8%)	1,038 (50.3%)	420 (50.5%)	
Ever	1,597 (12.8%)	207 (10.0%)	110 (13.2%)	
Current	3,934 (31.4%)	817 (39.6%)	302 (36.3%)	
Alcohol				0.01
More than once a month	3,283 (26.2%)	511 (24.8%)	201 (24.2%)	
Less than once a month	941 (7.5%)	133 (6.5%)	45 (5.4%)
Never	8,286 (66.2%)	1,418 (68.8%)	586 (70.4%)	
Peak expiratory flow (L/min)	288.6 ± 119.5	233.8 ± 107.1	193.0 ± 105.3	<0.01
Night sleep duration				<0.01
<360 min	4,138 (33.1%)	816 (39.6%)	347 (41.7%)	
360–419 min	2,699 (21.6%)	410 (19.9%)	139 (16.7%)	
420–479 min	2,196 (17.6%)	271 (13.1%)	98 (11.8%)	
480–539 min	2,416 (19.3%)	374 (18.1%)	149 (17.9%)	
≥540 min	1,061 (8.5%)	191 (9.3%)	99 (11.9%)	
Mean duration(h)	6.2 ± 1.9	6.0 ± 2.2	6.0 ± 2.3	<0.01
Napping duration				<0.01
0 min	4,916 (39.3%)	1,022 (49.6%)	386 (46.4%)	
1–59 min	2,207 (17.6%)	306 (14.8%)	120 (14.4%)	
60–119 min	3,651 (29.2%)	479 (23.2%)	206 (24.8%)	
≥120 min	1736 (13.9%)	255 (12.4%)	120 (14.4%)	
Accident				0.32
No	11,728 (93.7%)	1928 (93.5%)	790 (95.0%)
Yes	782 (6.3%)	134 (6.5%)	42 (5.0%)
Fallen down				<0.01
No	10,271 (82.1%)	1,666 (80.8%)	646 (77.6%)	
Yes	2,239 (17.9%)	396 (19.2%)	186 (22.4%)	
Hip fracture				0.07
No	12,326 (98.5%)	2025 (98.2%)	812 (97.6%)	
Yes	184 (1.5%)	37 (1.8%)	20 (2.4%)	
Depression (CED-S10≥12)				<0.01
No	9,094 (72.7%)	1,347 (65.3%)	545 (65.5%)	
Yes	3,416 (27.3%)	715 (34.7%)	287 (34.5%)	
Mean scores	8.9 ± 5.1	9.7 ± 5.4	9.8 ± 5.7	<0.01
Comorbidity profiles				<0.01
Class 1	1956 (15.6%)	169 (8.2%)	78 (9.4%)	
Class 2	560 (4.5%)	124 (6.0%)	56 (6.7%)	
Class 3	8,511 (68.0%)	1,506 (73.0%)	595 (71.5%)	
Class 4	1,483 (11.9%)	263 (12.8%)	103 (12.4%)	

Note: Class 1: dominant heart diseases or risks; Class 2: dominant chronic lung diseases; Class 3: minimal or least diseases; and Class 4: dominant digestive diseases and rheumatism.

**FIGURE 1:**
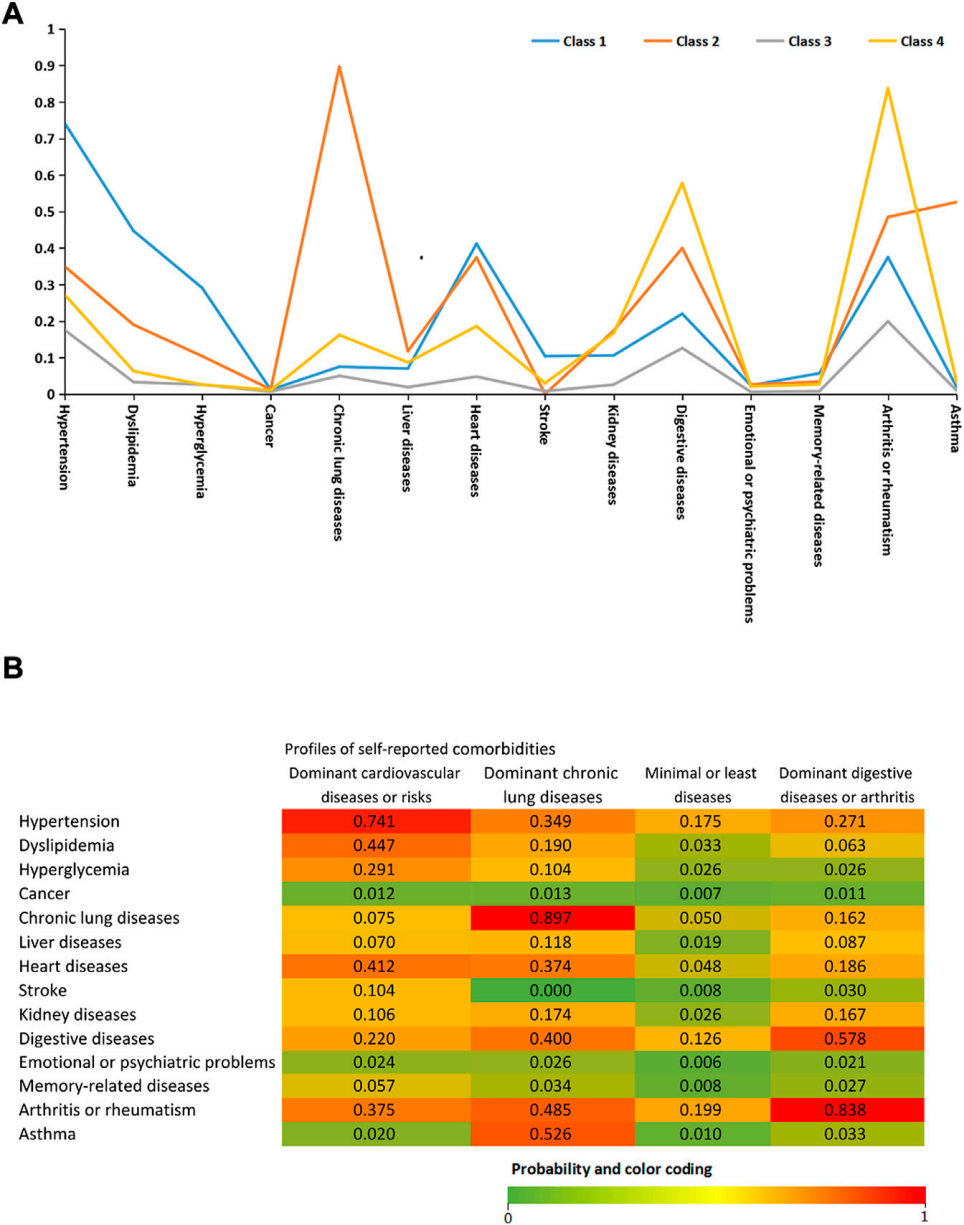
**(A)** Latent class analysis of 14 doctor-diagnosed chronic diseases. Class 1: dominant heart diseases or risks (reference; *n* = 2,203 [14.3%]). Class 2: dominant chronic lung diseases (n = 740 [4.8%]). Class 3: minimal or least diseases (*n* = 10,612 [68.8%]). Class 4: dominant digestive diseases and rheumatism (*n* = 1849 [12.1%]) **(B)**Probability of each indicator variable across the four comorbidity profiles.

Based on the 14 chronic diseases, LCA delineated four classes as the best-fit LCA model in our study population (see Figures 1A, B). Four latent profiles were named as dominant heart diseases or risks (including hypertension, dyslipidemia, hyperglycemia, and heart diseases, class 1), dominant chronic lung diseases (chronic lung diseases and asthma, class 2), minimal or least diseases (class 3, reference), and dominant digestive diseases and rheumatism (class 4). Supplementary Table S1 demonstrates the characteristics of the study population stratified by comorbidity profiles in the CHARLS. The proportion of the four comorbidity profiles was 14.3% (N = 2,203), 4.8% (N = 740), 68.8% (N = 10,612), and 12.1% (N = 1849), respectively. Compared with the minimal or least disease profiles (class 3), the decreased mean value of PEF was shown in class 2 (−55.3 L/min, 95% CI: 66.2 L/min, and −44.5 L/min) and class 4 (−12.8 L/min, 95% CI: 20.1 L/min, and −5.7 L/min) but not in class 1. Meanwhile, the scores of CED-S10 significantly increased by 0.4 scores (95% CI: 0.2, 0.7) in class 1, 0.9 scores (95% CI: 0.4, 1.4) in class 2, and 1.2 scores (95% CI: 1.0, 1.6) in class 3 than class 4.

The dominant chronic lung disease profile had a significantly lower BMI (23 ± 4.1 kg/m^2^ vs. 24.8 ± 4.2 kg/m^2^ vs., *p* < 0.001) and PEF (224.6 ± 125.6 vs. 282.5 L/min ± 119.5 L/min, *p* < 0.01), a higher proportion of underweight (10.5% vs. 4.6%, *p* < 0.001) and older individuals aged ≥80 years (6.4% vs. 4.6%, *p* = 0.002), and a lower proportion of obesity (10.5% vs. 19.6%, *p* < 0.001) compared with class 1. In the four comorbidity profiles, the highest prevalence of sarcopenia and severe sarcopenia were shown in class 2 (24.3% and 7.6%), followed by class 3 (19.8% and 5.6%), class 4 (19.8% and 5.6%), and class 1 (11.2% and 3.5%). Compared with the reference group (class 3), the multivariate-adjusted ORs (95% CIs) of sarcopenia in model 3 were 0.72 (0.60–0.88) for class 1, 1.17 (0.92–1.51) for class 2, and 0.92 (0.77–1.09) for class 4. Multivariate-adjusted ORs indicated that the prevalence of severe sarcopenia among four comorbidity profiles had no significant difference in all three models (see Table 2).

**TABLE 2:** Associations between sarcopenia with comorbidity profiles and napping duration in the China Health and Retirement Longitudinal Study.

	Model 1	Model 2	Model 3
Associations between sarcopenia with comorbidity profiles and napping duration			
Comorbidity profiles			
Class 1	0.73 (0.60, 0.88)*	0.73 (0.60, 0.88)*	0.72 (0.60, 0.88)*
Class 2	1.44 (1.13, 1.84)*	1.20 (0.93, 1.54)	1.17 (0.92, 1.51)
Class 3	Ref	Ref	Ref
Class 4	0.95 (0.81, 1.13)	0.93 (0.79, 1.11)	0.92 (0.77, 1.09)
Napping duration			
0 min	Ref	Ref	Ref
1–59 min	0.77 (0.65, 0.91)*	0.79 (0.67, 0.93)*	0.80 (0.68, 0.94)*
60–119 min	0.80 (0.70, 0.92)*	0.83 (0.72, 0.95)*	0.83 (0.72, 0.96)*
120 min	0.89 (0.75, 1.05)	0.89 (0.74, 1.05)	0.89 (0.75, 1.06)
Associations between severe sarcopenia with comorbidity profiles and napping duration			
Comorbidity profiles			
Class 1	0.96 (0.74, 1.25)	0.98 (0.75, 1.27)	0.97 (0.74, 1.27)
Class 2	1.34 (0.97, 1.84)	1.02 (0.74, 1.41)	1.01 (0.73, 1.39)
Class 3	Ref	Ref	Ref
Class 4	1.05 (0.83, 1.34)	1.04 (0.81, 1.32)	1.02 (0.80, 1.30)
Napping duration			
0 min	Ref	Ref	Ref
1–59 min	0.85 (0.67, 1.08)	0.85 (0.67, 1.07)	0.85 (0.67, 1.07)
60–119 min	0.94 (0.78, 1.14)	0.97 (0.80, 1.18)	0.97 (0.80, 1.18)
120 min	1.06 (0.84, 1.35)	1.04 (0.82, 1.33)	1.05 (0.82, 1.33)

Model 1 adjusted the following variables: sex, age, region, urban/rural, married status, and body mass index. Model 2 adjusted the following variables: sex, age, region, urban/rural, education levels, married status, body mass index, smoking, alcohol, night sleep duration, and peak expiratory flow. Model 3 included the following variables: sex, age, region, urban/rural, education levels, married status, smoking, alcohol, body mass index, smoking, alcohol, night sleep duration, peak expiratory flow, accident, fallen down, hip fracture, depression, napping Duration, and comorbidity profiles. Class 1: dominant heart diseases or risks; Class 2: dominant chronic lung diseases; Class 3: minimal or least diseases; and Class 4: dominant digestive diseases and rheumatism^#^ *p* < 0.05; *p < 0.01

**FIGURE 2:**
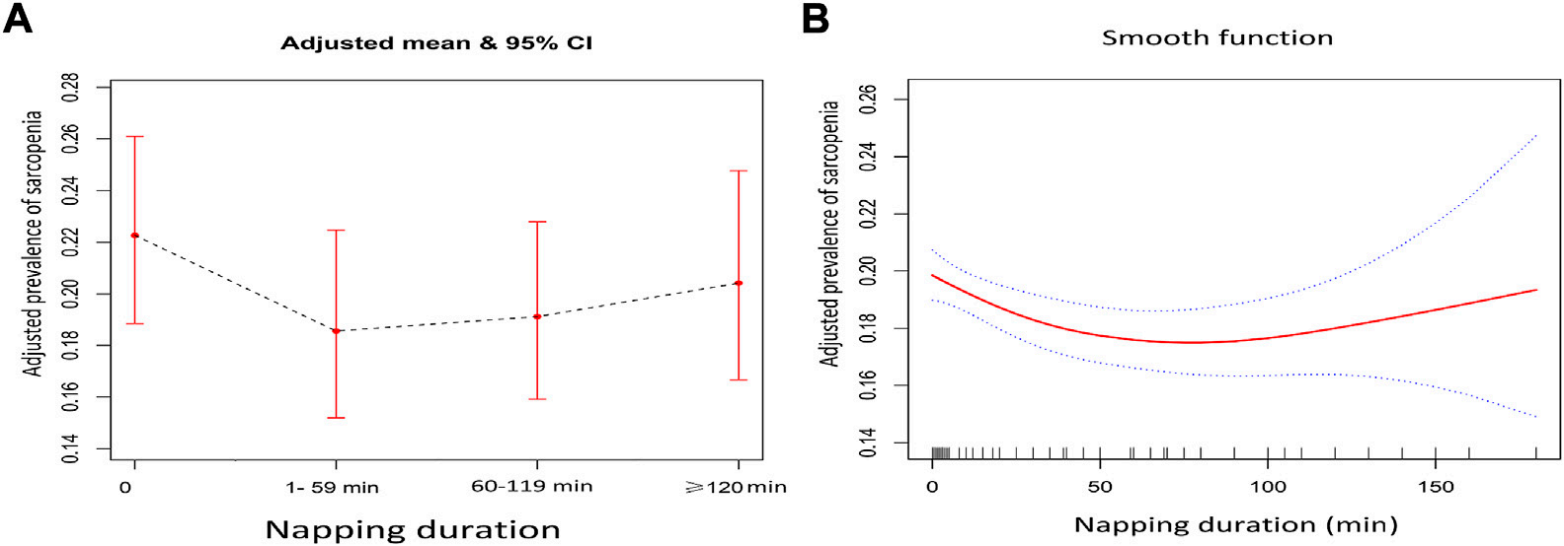
**(A)** Adjusted prevalence of the four daytime napping groups in model 3 when daytime napping was considered as a categorical variable and **(B)** adjusted prevalence of different daytime napping in model 3 when daytime napping was considered as a continuous variable.

**FIGURE 3:**
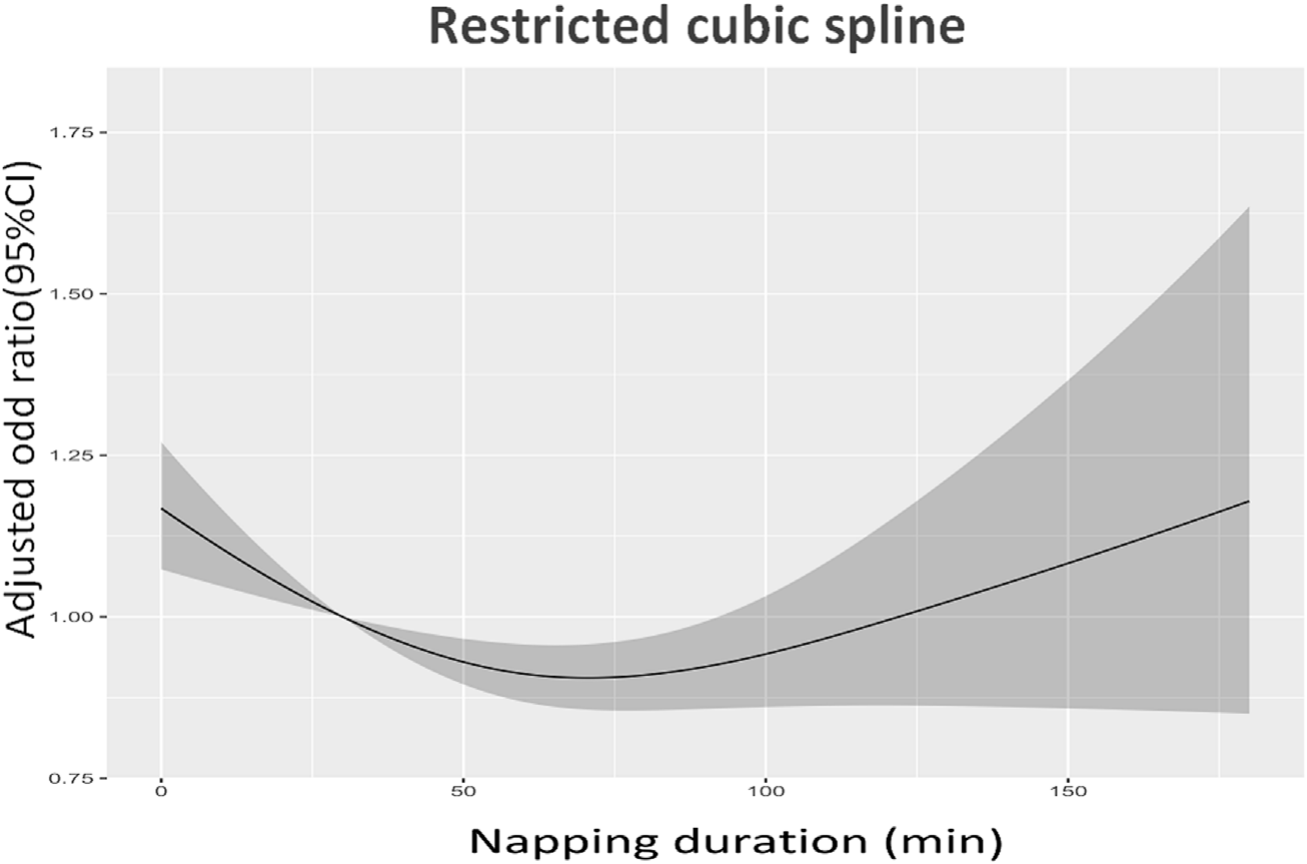
Restricted cubic spline curves about the association between daytime napping and the prevalence of sarcopenia in model 3.

The highest prevalence of sarcopenia and severe sarcopenia was shown in individuals who did not nap (22.3% and 6.1%), following by those napped for ≥120 min (17.8% and 5.7%), 60–119 min (16.2% and 4.6%), and 1–59 min (15.8% and 4.8%). Figures 2A, B demonstrate the association between daytime napping and the prevalence of sarcopenia in model 3. The multivariate-adjusted ORs suggested that individuals who napped for 1–59 min (adjusted OR = 0.80, 95% CI: 0.68–0.94, *p* < 0.01, and in model 3) and 60–119 min (adjusted OR = 0.83, 95% CI: 0.72–0.96, and *p* < 0.01, in model 3) but not ≥120 min (adjusted OR = 0.89, 95% CI: 0.75–1.06, *p* = 0.21, and in model 3) were associated with significantly lower prevalence of sarcopenia compared with those with no napping. Restricted cubic spline curves showed two reference nodes (30 and 120 min) and suggested that individuals who napped about 60 min seemingly had the lowest risk of sarcopenia (see Figures 2B, 3). When stratified by comorbidity profiles, the highest prevalence of sarcopenia in all four napping groups was shown in dominant chronic lung disease profile. Multivariate-adjusted ORs suggested that daytime napping duration had no significant correlation with the prevalence of severe sarcopenia in all three models (see Table 2).

## Discussion

This population-based study approved two hypotheses initially formulated. The dominant chronic lung disease profile had the highest prevalence of sarcopenia, followed by the minimal or least disease profile, the dominant digestive diseases and rheumatism profile, and dominant heart diseases or risk profile. Our study also suggested that the associations between daytime napping with sarcopenia and severe sarcopenia showed a J-sharp with optimal napping duration of 60 min. Both insufficient and excessive daytime napping durations were associated with the increased prevalence of sarcopenia in the Chinese older population. In addition, we also found that the prevalence and severity of sarcopenia had the relationships with aging, PEF, and the scores of depressive symptoms.

Increasing recognition suggested that sarcopenia is not just a primary or age-related disorder but also considered a secondary disorder caused by underlying diseases such as chronic obstructive pulmonary disease (COPD) or other conditions (Benz et al., 2019). Inflammation cytokines, stress, and microvascular changes are deemed to play an important role in the development of sarcopenia although the pathophysiology of sarcopenia has not been fully elucidated (Cruz-Jentoft and Sayer, 2019). Previous results identified that aging is an independent risk factor of sarcopenia (Byun et al., 2017; Chen et al., 2021). Our study found that sarcopenia and its severity were significantly associated with aging. Previous studies recommended that PEF with cut-offs of 200 L/min (Marco et al., 2020) and 300 L/min (Kera et al., 2018) may be a potentially useful screening tool of sarcopenia in clinical practice. Studies involving 2,422 older Indonesians demonstrated that the peak expiratory flow rates (PEFRs) of <50% and 50%–80% are associated with nearly 5.22 and 1.88 times increased risk of sarcopenia than PEFRs of ≥80% (Ridwan et al., 2021). Our study further identified that the value of PEF was negatively correlated with its severity of sarcopenia. PEF measurement needs to use maximal muscle power in an effort-dependent manner and may reflect the strength of abdominal and intercostal muscles as well as the elastic recoil of the lung and chest wall (Yamada et al., 2016). Decreased PEF means the weakness of muscle strength and muscle dysfunction manifested by muscle fiber-type switching (Yamada et al., 2016). These results indicated that monitoring PEF has an important value for the prevention, diagnosis, and management of sarcopenia. Wang et al. (2021) found that the prevalence of sarcopenia shows a significant association with depressive symptoms measured by the five-item CES-D. Two other studies also observed that the higher scores on the CES-D are related to the higher prevalence of sarcopenia in older individuals (Kim et al., 2011; Kurita et al., 2021). Our study suggested that depressive symptoms measured by the 10-item CES-D are positively associated with the prevalence of sarcopenia and severe sarcopenia. The decline of social activity secondary to depression might result in sarcopenia. Chronic low-grade inflammation and oxidative stress caused by depression were also the common molecule-driven pathways of developing sarcopenia (Szlejf et al., 2019). These studies indicated that the assessment of depressive symptoms based on CES-D may be an effective tool of screening the risk of sarcopenia.

Meta-analysis found that the overall prevalence of sarcopenia in individuals with COPD was two times higher than the healthy elderly population (Benz et al., 2019). Chen and colleagues observed that COPD had the biggest contribution in developing severe sarcopenia among six disorders for male older adults (Chen et al., 2021). Our study determined four comorbidity profiles on the basis of 14 chronic diseases using latent class analysis and demonstrated that the dominant chronic lung disease profile was associated with the highest risk of sarcopenia and severe sarcopenia among four comorbidity profiles. Compared with the dominant heart diseases or risk profile, the prevalence of sarcopenia increased by nearly 62% in the dominant chronic lung disease profile. Physical inactivity, oxidative stress, inflammatory cytokines (such as IL-6 and TNF-α), reduced microvascular flow to muscles, and inadequate energy intake were regarded as common features linked with chronic lung diseases and sarcopenia (Benz et al., 2019). In addition, it is well-known that severe COPD can lead to limb-muscle dysfunction by a shift from type I to II fibers, which may contribute to the loss of muscle strength (Maltais et al., 2014). The lowest value of PEF was shown in the dominant chronic lung disease profile which potentially limited the ability of physical activity. Individuals in the dominant heart diseases or risks profile had the highest proportion of overweight (35.6%) and obesity (19.7%) among the four comorbidity profiles, which may be the main cause of low prevalence of sarcopenia.

Sleep health may play an important role in muscle protein synthesis and degradation (Piovezan et al., 2015). Sleep problems are likely to affect muscle fiber loss and give rise to strength and function decline by suppressing anabolic hormone cascades and promoting catabolic pathways in the skeletal muscle (Piovezan et al., 2015). Hormonal and muscle metabolism imbalances, systemic inflammation, and insulin resistance secondary to sleep deprivation may contribute to the development of sarcopenia (Prokopidis and Dionyssiotis, 2021). Furthermore, protein and metabolite-level analyses clearly demonstrated that acute sleep deprivation was associated with downregulated protein levels in the glycolysis pathway and molecular signature change of muscle breakdown in the skeletal muscle, which may potentially promote sarcopenia (Cedernaes et al., 2018). A 3-year longitudinal cohort study (Han et al., 2022) and the meta-analysis involving five cross-sectional studies (Pourmotabbed et al., 2020) both suggested that sleep-duration reduction is associated with the increased risk of sarcopenia. Daytime napping is deemed as a potential and powerful “public health tool” to counteract the negative short- and long-term consequences caused by sleep problems (Faraut et al., 2017). Our findings provided a new evidence-based medicine for this viewpoint in clinical practice. For older Chinese adults, smooth function and restricted cubic spline curves both indicated that 60 min as the optimal daytime napping were associated with significantly decreased risk of sarcopenia. We speculated that the possible mechanisms linked with sarcopenia and napping mainly include the pathways of pathophysiology and physical performance. The inflammation, oxidative stress, and microvascular impairment were three crucial pathophysiological causes of developing sarcopenia (Cruz-Jentoft and Sayer, 2019). The current studies suggested that optimal daytime napping can lead to stress-release effects secondary to sleep deprivation, decline immuno-inflammatory effects consecutive to sleep restriction, and ameliorate microvascular health (Faraut et al., 2017; Liu et al., 2022). These changes, secondary to optimal daytime napping, potentially reverse the pathophysiological causes of developing sarcopenia. Physical performance was regarded as an important measure of the diagnosis and severity of sarcopenia. Daytime napping can improve the physical performance and reduce the muscular and oxidative damages caused by sleep deprivation (Romdhani et al., 2020). However, not all daytime napping durations provided clinical benefit in the prevalence of sarcopenia. We found that daytime napping <30 min or ≥120 min were associated with the increased risk of sarcopenia compared with 60 min. The beneficial effects of daytime napping on health conditions were presumed to have the correlation with sleep architecture and usually took place in nonrapid eye-movement (NREM) sleep dominated by parasympathetic activity (Liu et al., 2022). When napping duration is more than 60 min, individuals may have rapid eye-movement sleep dominated by sympathetic activity. Slow-wave sleep in NREM sleep (mainly including stages 3 and 4 of sleep) can inhibit hypothalamic–pituitary adrenal axis and decrease cortisol release and catecholamine production caused by sympathetic activity (Liu et al., 2022). Sleep architecture is usually in stages 1 and 2 of sleep for individuals who napped for <30 min, which has no significant benefit for health conditions due to the absence of slow-wave sleep. Therefore, insufficient and excessive daytime napping duration may result in adverse clinical outcomes. Our study provided a new recognition for the impact of daytime napping on health outcomes of older individuals.

Our study had the following strengths. A large-sample study population involving 15,404 older Chinese adults came from a nationally representative study that provided an ideal setting for studying napping habits and decreased the risk of selection bias secondary to little sample and local region. We determined four comorbidity profiles using a data-driven clustering technique (latent class analysis) and found the correlation between the comorbidity profiles with the prevalence of sarcopenia, which will provide a new direction in the precise prevention and control of sarcopenia. Various research methods were used to explore the relationship between daytime napping duration and the risk of sarcopenia which made our results more convincing.

The main limitation of this study was that the ASM was estimated by a validated anthropometric equation rather than dual X-ray absorptiometry (DXA) and bioelectrical impedance analysis (BIA). However, several studies suggested that this estimation formula for the ASM using anthropometric values was confirmed to harbor strong agreement with DXA in the Chinese population (Wen et al., 2011; Yang et al., 2017; Wu et al., 2021). For the large-sample epidemiological survey, using anthropometric equations to assess the ASM combined with gait speed and handgrip strength was a cost-effective alternative to DXA to improve the diagnosis of sarcopenia; meanwhile, it decreased the risk of radiation exposure secondary to DXA and had a high cost of measurements (Wu et al., 2021). Although we have adjusted many potentially confounding risks of sarcopenia, the absence of some data (such as physical activity) may lead to bias in the observed relations (Gao et al., 2022). In addition, self-report daytime napping duration and chronic disorders might exist in recall bias. Meanwhile, the lack of the frequency of daytime napping restricted us to further explore the association between daytime napping and sarcopenia. Finally, causal relationships between sarcopenia with daytime napping duration and comorbidity profiles could not be established owing to the cross-sectional nature of this study.

Our study suggests that the dominant chronic lung disease profile and insufficient and excessive daytime napping are associated with the increased risk of sarcopenia. This study might be the first important step to better understand the potential role of daytime napping and comorbidity profiles in the prevalence of sarcopenia. Our results underline that optimal napping duration and focus on monitoring a specific population may more efficiently prevent and reverse sarcopenia. Further large-sample longitudinal studies are warranted to explore whether the duration and frequency of objective daytime napping affect the risk of developing sarcopenia in different populations.

## Data Availability

The original contributions presented in the study are included in the article/Supplementary Material; further inquiries can be directed to the corresponding authors.
