# Supplementary material for: Daytime napping, comorbidity profiles, and the risk of sarcopenia in older individuals
Source: Front Physiol. 2022 Nov 1;13:1000593. doi: 10.3389/fphys.2022.1000593 (PMC9663836; doi:10.3389/fphys.2022.1000593)
Supplement: Supplementary file 1 [file DataSheet1.docx]

| Table S1: The characteristics of the study population stratified by comorbidity profiles in the China Health and Retirement Longitudinal Study | | | | | |
| --- | --- | --- | --- | --- | --- |
|  | Class 1 | Class 2 | Class 3 | Class 4 | *P* |
| N | 2203(14.3%) | 740(4.8%) | 10612(68.8%) | 1849(12.1%) |  |
| Sex |  |  |  |  | <0.01 |
| Male | 1030 (46.8%) | 456 (61.6%) | 5626 (53.0%) | 885 (47.9%) |  |
| Female | 1173 (53.2%) | 284 (38.4%) | 4986 (47.0%) | 964 (52.1%) |  |
| Age |  |  |  |  | <0.01 |
| 60-69 years | 1479 (67.1%) | 457 (61.8%) | 7117 (67.1%) | 1300 (70.3%) |  |
| 70-79 years | 622 (28.2%) | 236 (31.9%) | 2937 (27.7%) | 474 (25.6%) |  |
| ≥80 years | 102 (4.6%) | 47 (6.4%) | 558 (5.3%) | 75 (4.1%) |  |
| Mean age(year) | 67.6 ± 6.1 | 68.3 ± 6.2 | 67.6 ± 6.3 | 67.2 ± 5.9 | <0.01 |
| Region |  |  |  |  | <0.01 |
| Southwest | 533 (24.2%) | 238 (32.2%) | 3026 (28.5%) | 720 (38.9%) |  |
| South and central | 1059 (48.1%) | 341 (46.1%) | 5666 (53.4%) | 888 (48.0%) |  |
| North | 611 (27.7%) | 161 (21.8%) | 1920 (18.1%) | 241 (13.0%) |  |
| Urban/Rural |  |  |  |  | <0.01 |
| Rural | 1171 (53.2%) | 471 (63.6%) | 6733 (63.4%) | 1274 (68.9%) |  |
| Urban | 1032 (46.8%) | 269 (36.4%) | 3879 (36.6%) | 575 (31.1%) |  |
| Married status |  |  |  |  | 0.63 |
| Current unmarried | 431 (19.6%) | 140 (18.9%) | 1969 (18.6%) | 334 (18.1%) |  |
| Current married | 1772 (80.4%) | 600 (81.1%) | 8643 (81.4%) | 1515 (81.9%) |  |
| Body mass index category(BMI,kg/m2) | | |  |  | <0.01 |
| Underweight | 102 (4.6%) | 78 (10.5%) | 909 (8.6%) | 165 (8.9%) |  |
| Normal | 883 (40.1%) | 374 (50.5%) | 5799 (54.6%) | 1001 (54.1%) |  |
| Overweight | 787 (35.7%) | 210 (28.4%) | 2960 (27.9%) | 509 (27.5%) |  |
| Obesity | 431 (19.6%) | 78 (10.5%) | 944 (8.9%) | 174 (9.4%) |  |
| Mean BMI | 24.8 ± 4.2 | 23.0 ± 4.1 | 23.1 ± 4.3 | 23.1 ± 3.7 | <0.01 |
| Smoking |  |  |  |  | <0.01 |
| Never | 1326 (60.2%) | 320 (43.2%) | 5769 (54.4%) | 1022 (55.3%) |  |
| Ever | 283 (12.8%) | 155 (20.9%) | 1274 (12.0%) | 202 (10.9%) |  |
| Current | 594 (27.0%) | 265 (35.8%) | 3569 (33.6%) | 625 (33.8%) |  |
| Alcohol |  |  |  |  | <0.01 |
| More than once  a month | 462 (21.0%) | 170 (23.0%) | 2901 (27.3%) | 462 (25.0%) |  |
| Less than once a month | 175 (7.9%) | 41 (5.5%) | 785 (7.4%) | 118 (6.4%) |  |
| Never | 1566 (71.1%) | 529 (71.5%) | 6926 (65.3%) | 1269 (68.6%) |  |
| PEF(L/min) | 282.8 ± 119.5 | 224.6 ± 125.6 | 279.9 ± 120.0 | 267.1 ± 115.1 | <0.01 |
| Night sleep duration |  |  |  |  | <0.01 |
| <360 min | 798 (36.2%) | 307 (41.5%) | 3438 (32.4%) | 758 (41.0%) |  |
| 360-419 min | 477 (21.7%) | 131 (17.7%) | 2258 (21.3%) | 382 (20.7%) |  |
| 420-479 min | 345 (15.7%) | 105 (14.2%) | 1842 (17.4%) | 273 (14.8%) |  |
| 480-539 min | 405 (18.4%) | 131 (17.7%) | 2102 (19.8%) | 301 (16.3%) |  |
| ≥540 min | 178 (8.1%) | 66 (8.9%) | 972 (9.2%) | 135 (7.3%) |  |
| Mean duration | 6.1 ± 2.0 | 6.0 ± 2.2 | 6.3 ± 2.0 | 5.9 ± 2.1 | <0.01 |
| Napping duration |  |  |  |  | <0.01 |
| 0 min | 774 (35.1%) | 280 (37.8%) | 4419 (41.6%) | 851 (46.0%) |  |
| 1-59 min | 444 (20.2%) | 128 (17.3%) | 1772 (16.7%) | 289 (15.6%) |  |
| 60-119 min | 696 (31.6%) | 221 (29.9%) | 2948 (27.8%) | 471 (25.5%) |  |
| ≥120 min | 289 (13.1%) | 111 (15.0%) | 1473 (13.9%) | 238 (12.9%) |  |
| Accident |  |  |  |  | 0.01 |
| No | 2088 (94.8%) | 677 (91.5%) | 9970 (94.0%) | 1711 (92.5%) |  |
| Yes | 115 (5.2%) | 63 (8.5%) | 642 (6.0%) | 138 (7.5%) |  |
| Fallen down |  |  |  |  | <0.01 |
| No | 1779 (80.8%) | 587 (79.3%) | 8802 (82.9%) | 1415 (76.5%) |  |
| Yes | 424 (19.2%) | 153 (20.7%) | 1810 (17.1%) | 434 (23.5%) |  |
| Hip fracture |  |  |  |  | 0.03 |
| No | 2178 (98.9%) | 724 (97.8%) | 10431 (98.3%) | 1830 (99.0%) |  |
| Yes | 25 (1.1%) | 16 (2.2%) | 181 (1.7%) | 19 (1.0%) |  |
| Depression |  |  |  |  | <0.01 |
| No | 1535 (69.7%) | 484 (65.4%) | 7807 (73.6%) | 1160 (62.7%) |  |
| Yes | 668 (30.3%) | 256 (34.6%) | 2805 (26.4%) | 689 (37.3%) |  |
| Mean scores | 9.2 ± 5.2 | 9.7 ± 5.3 | 8.8 ± 5.2 | 10.0 ± 5.5 | <0.01 |
| Sarcopenia |  |  |  |  | <0.01 |
| No | 1956 (88.8%) | 560 (75.7%) | 8511 (80.2%) | 1483 (80.2%) |  |
| Yes | 247 (11.2%) | 180 (24.3%) | 2101 (19.8%) | 366 (19.8%) |  |
| Severe sarcopenia |  |  |  |  | <0.01 |
| No | 2125 (96.5%) | 684 (92.4%) | 10017 (94.4%) | 1746 (94.4%) |  |
| Yes | 78 (3.5%) | 56 (7.6%) | 595 (5.6%) | 103 (5.6%) |  |
| Note: Class 1: dominant heart diseases or risks ; Class 2: dominant chronic lung diseases; Class 3: minimal or least diseases; Class 4: dominant digestive diseases and rheumatism | | | | | |


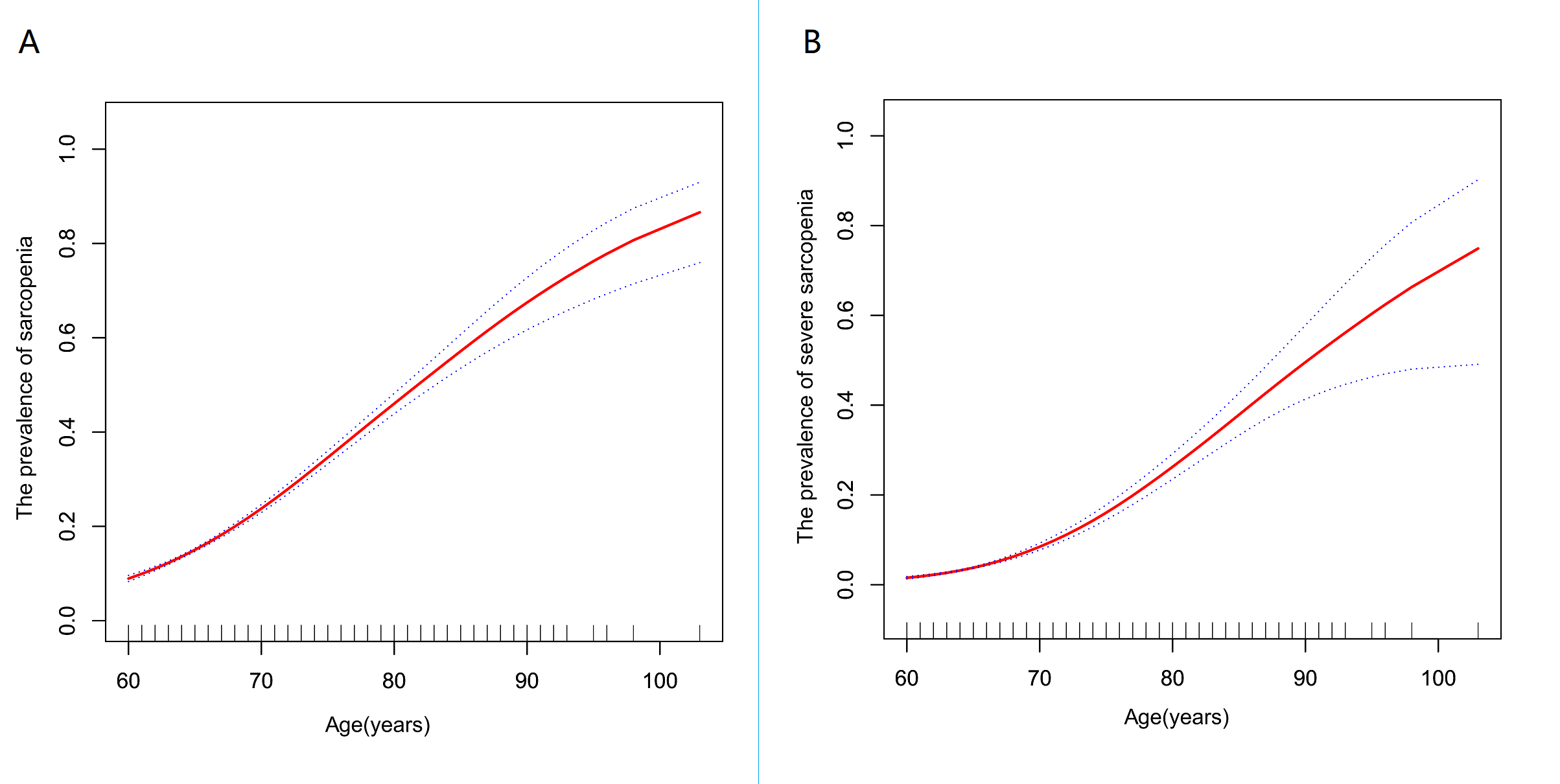


S1.Fig: (A) Association between aging and the prevalence of sarcopenia; (B) Association between aging and the prevalence of severe sarcopenia.


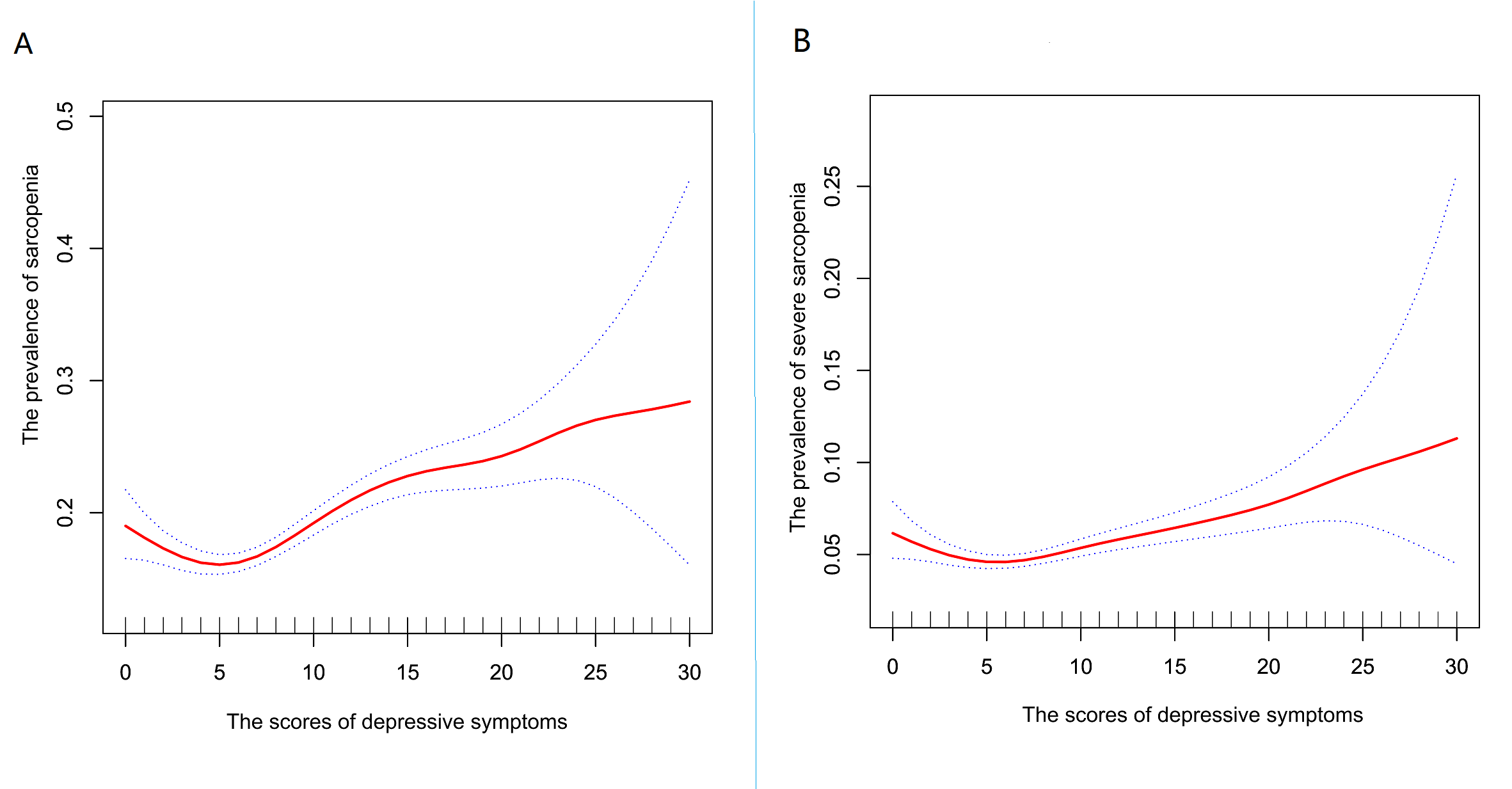


S2.Fig: (A) Association between the scores of depressive symptoms and the prevalence of sarcopenia; (B) Association between the scores of depressive symptoms and the prevalence of severe sarcopenia.
